# A Qualitative Study of Service Provision for Alcohol Related Health Issues in Mid to Later Life

**DOI:** 10.1371/journal.pone.0148601

**Published:** 2016-02-05

**Authors:** Catherine Haighton, Graeme Wilson, Jonathan Ling, Karen McCabe, Ann Crosland, Eileen Kaner

**Affiliations:** 1 Institute of Health and Society, Newcastle University, Newcastle upon Tyne, United Kingdom; 2 Department of Pharmacy, Health and Well-being, Sunderland University, Sunderland, United Kingdom; Penn State College of Medicine, UNITED STATES

## Abstract

**Aims:**

Epidemiological surveys over the last 20 years show a steady increase in the amount of alcohol consumed by older age groups. Physiological changes and an increased likelihood of health problems and medication use make older people more likely than younger age groups to suffer negative consequences of alcohol consumption, often at lower levels. However, health services targeting excessive drinking tend to be aimed at younger age groups. The aim of this study was to gain an in-depth understanding of experiences of, and attitudes towards, support for alcohol related health issues in people aged 50 and over.

**Methods:**

Qualitative interviews (n = 24, 12 male/12 female, ages 51–90 years) and focus groups (n = 27, 6 male/21 female, ages 50–95 years) were carried out with a purposive sample of participants who consumed alcohol or had been dependent.

**Findings:**

Participants’ alcohol misuse was often covert, isolated and carefully regulated. Participants tended to look first to their General Practitioner for help with alcohol. Detoxification courses had been found effective for dependent participants but only in the short term; rehabilitation facilities were appreciated but seen as difficult to access. Activities, informal groups and drop-in centres were endorsed. It was seen as difficult to secure treatment for alcohol and mental health problems together. Barriers to seeking help included functioning at a high level, concern about losing positive aspects of drinking, perceived stigma, service orientation to younger people, and fatalistic attitudes to help-seeking. Facilitators included concern about risk of fatal illness or pressure from significant people.

**Conclusion:**

Primary care professionals need training on improving the detection and treatment of alcohol problems among older people. There is also a compelling need to ensure that aftercare is in place to prevent relapse. Strong preferences were expressed for support to be provided by those who had experienced alcohol problems themselves.

## Introduction

Population ageing is taking place in nearly all the countries of the world and this is expected to continue over the coming years [1]. Alcohol problems can accumulate in mid to later life and are associated with social, psychological, physical and economic consequences[2]. Alcohol is a risk factor for coronary heart disease, stroke, high blood pressure, cancers, pancreatitis and liver cirrhosis[3]. However as people age, physiological changes mean that that older people are more sensitive to the effects of alcohol, and experience problems at lower levels of consumption[4][5] Because the average person aged 50 or above can be taking at least four prescribed medications a day[6] and alcohol is a major contraindication for many of these drugs, alcohol and medication interactions are common[7]. Combined alcohol and medication use is estimated to affect up to 19% of older Americans[8] while drinking alcohol for medicinal purposes is also prevalent[9][10].

Alcohol consumption has been associated with impairments in the instrumental activities of daily living[11] and can contribute to the onset of dementia and other age-related cognitive deficits[12], Parkinson’s disease and a range of psychological problems including depression and anxiety[13] Alcohol use is implicated in one-third of all suicides in the older population[14]. One in five older men and one in ten older women are drinking at harmful levels and these figures have increased by 40% and 100% respectively over the past 20 years[15]. However in mid to later life, alcohol problems are often misdiagnosed, under-detected and under-reported[16][17]. The ageing of populations worldwide means that the absolute number of older people with alcohol problems is increasing and a real danger exists that a “silent epidemic” may be evolving[16].

Recent research has suggested that age-specific practices required to meet the needs of older people, in relation to alcohol consumption, and draw them into treatment are poorly understood[18]. However research on alcohol consumption in older adults is still relatively scarce, there are notably few quantitative studies and no qualitative studies investigating support for alcohol related health issues in older people[9][10][19–21]. Therefore this research contributes to the limited body of in-depth qualitative evidence regarding the issues surrounding alcohol consumption in mid to later life. This paper is novel in aiming to gain an in-depth understanding of experiences of, and attitudes towards, support for alcohol related health issues in mid to later life. This has allowed recommendations to be made for future service provision tailored to this age group.

Mid to later life has been defined as individuals aged 50 and over to reflect the eligibility criteria of the UKs leading charity for older people. The study[22] was based in an urban area in North East England, a region with an older population and high rates of heavy drinking.

## Methods

The study involved qualitative interviews and focus groups with a purposive sample of middle aged and older people. Ethical approval was issued by Newcastle University Research Ethics Committee (application no. 000224/2009). Participants provided written informed consent to participate in this study. In line with the terms of consent to which participants agreed, the data are not publicly available and are not available to be shared outside the project team.

Twenty-four in-depth interviews (12 male, 12 female) were conducted between 19/11/09 and 15/03/10. Purposive sampling (a non-random method of ensuring that particular categories of cases within a sampling universe are represented in the final sample)[23] aimed to recruit both genders and represent a broad range of ages and self-reported drinking practices and was intended to reflect those who might request help or support from the UKs leading charity for older people. Three branches of a national charity aiming to improve later life (Age UK) and two services for alcohol problems covering a wide geographical area distributed research information leaflets to clients aged 50 and over with experience of drinking alcohol. Staff members from the recruiting organisations invited clients to consider participating in an interview, answered any questions they had about the research and asked those who were interested to complete a consent form. All potential participants were contacted by telephone by one of the authors (GW) to arrange an interview. As the initial sample appeared to consist of a large proportion of participants who described themselves as recovering dependent drinkers, strategic ‘snowballing’ was used to add further interviewees. This involved existing study subjects recruiting future subjects, who were not using services and therefore not recovering dependent drinkers, from among their acquaintances. Considerable diversity was ultimately achieved (see Table 1).

**Table 1:** Interviewee characteristics.

Interviewee number	Age	Gender	From interview: self-reported drinking status /behavior	From interview: lives with
1	61	m	Recovering dependent drinker^a^ abstinent for 2.5 years	Other residents
2^b^	59	f	Recovering dependent drinker sensible drinker^c^ for 12 years	Adult child, adult child‘s partner, grandchild
3^a^	56	f	Dependent drinker^d^	Husband, adult child
4^a^	61	m	Dependent drinker	Alone
5	52	m	Recovering dependent drinker abstinent for 2 months	Alone
6	59	m	Recovering dependent drinker abstinent for 4 weeks	Wife
7	57	m	Recovering dependent drinker abstinent for 2 years	Wife
8^a^	74	m	3 litres whisky per week	Alone
9	62	m	Previously 3–4 pints on 3–4 nights per week abstinent for 6 months	Alone
10	60	m	Recovering dependent drinker abstinent for 1 year	Alone
11	55	f	Recovering dependent drinker abstinent for 9 weeks	Alone
12	51	f	Previously 3 litres cider + 2 cans per day abstinent for 1 year	Husband, teenage children
13	68	m	Recovering dependent drinker abstinent for 5 years	Unknown
14^a^	58	f	Previously 2 bottles spirits per weekend reduced to occasional glass of wine for past 2 years	Alone
15^a^	65	m	Previously 13 pints beer per night reduced to 2–3 pints per night for 1.5 years	Alone
16^a^	52	f	Reducing dependent drinker from bottles of spirits to 4 pints, 5 days a week	Husband, adult children
17^a^	70	f	Bottle of wine a day abstinent while hospitalised only	Other residents
18^a^	78	f	Occasional minimal drinker	Other residents
19^a^	83	f	Occasional minimal drinker	Other residents
20^a^	90	f	Occasional minimal drinker	Other residents
21^a^	56	m	4–5 pints/night, 2 nights/week reduced from previous levels	Partner & sons
22^a^	59	f	Previously a bottle a night for a period reduced to glass or two of wine a night, not every night	Partner
23^a^	58	F	4 vodka & tonics a night, twice a week	Partner
24^a^	72	M	4 pints beer every night, sometimes two gin and tonics	Wife

^a^ Recovering dependent drinker defined as meeting the diagnostic criteria for full remission of alcohol dependence[24]

^b^ Currently consuming alcohol

^c^ Sensible drinker defined as drinking within the governments recommended limits for sensible alcohol consumption at the time of interview (men no more than three to four units of alcohol per day/women no more than two to three units of alcohol per day)[25]

^d^ Dependent drinker defined as psychiatric diagnosis in which an individual is physically or psychologically dependent upon drinking alcohol[26]

To compare individual accounts with socially negotiated versions of drinking in mid to later life, three focus groups were facilitated between 15/03/10 and 19/10/10. Staff at the three branches of Age UK distributed research information leaflets to members with experience of drinking alcohol and invited them to consider participating in a focus group, answered any questions they had about the research and asked those who were interested to complete a consent form. The first group comprised 9 participants (1 male, 8 female, ages 79–95); the second group comprised 12 participants (5 male, 7 female, ages 50–85) and the third comprised 6 participants (all female, ages 51–76). To encourage participation, focus group participants were not required to disclose personal details other than age and date of birth; these data were gathered on consent forms. At the groups, participants were invited by the facilitator (GW) to offer views in general rather than recounting personal experience in front of others.

Interviews and focus groups were conducted by GW, an experienced post-doctoral researcher, and lasted between 40 and 150 minutes either at individual respondent’s homes or the offices of participating organisations. The research team prepared topic guides (available on request) to initiate or return discussion to the research topics. Interview and focus group data were audio recorded, transcribed verbatim, anonymised and loaded into NVivo qualitative software, version 10[27]. Data were analysed using a grounded approach to identify axial codes [28–29] and involved repeatedly reading transcripts and identifying emerging codes; early analysis informed later interviews and focus groups. Codes were refined through discussion amongst the authors with consideration of deviant cases in order to provide a full account of participants’ views. Focus group data were used to triangulate findings from individual interviews. In the results section below we report findings from individual interviews, then consider how the focus group data inform or challenge these codes. This paper is not exhaustive in its presentation of the analysis rather it focuses on specific codes with each subheading representing a distinct code emerging from the data relating to service provision and use.

## Results

### Drinking in mid to later life

Nine of 15 participants still drinking alcohol described themselves as currently drinking sensibly, having reduced their alcohol consumption from previously higher levels. However some individuals had not reduced their drinking. The heaviest drinker reported consuming nine pints and a bottle of wine most days. Most women who reported drinking little nevertheless felt they had increased their consumption as they entered mid to later life.

Going out to drink was a feature of the accounts of younger interviewees (50–69 years), with special occasions associated with heavier drinking:

I’ve had the nights out with the girls, where we’ve got all of our troubles over in the last half hour and then concentrated on having a few good drinks and all that. I’ve had that, done that, been there, thoroughly enjoyed it, liked the feeling that it gave me. (22; female, 59yrs)

Choice of where to drink was limited for interviewees aged 70+ by their mobility or other circumstances, for instance if in a care home. Two of the older interviewees described being influenced to drink more by people living close to them. One older woman found she started to drink more when she became less mobile and received frequent visits at home from a neighbour who drank heavily.

Interviewees who reported dependence described their drinking in mid to later life as increasingly taking place alone and at home. For female dependent drinkers this was a consequence of being at home much of the time; male dependent drinkers associated starting to drink at home with a shift to using alcohol as a coping strategy, or finding that drinking with other people inhibited their drinking. However, some dependent drinkers of both sexes valued social drinking as being less boring than drinking in the house alone. Some problem drinkers described drinking as a continuous process through the day, saving something at the end of an evening for an ‘eye-opener’ the next morning:

I’ll come down on a morning after I’ve taken a bath upstairs. I hardly ever touch the drink upstairs, I’ll bring it down. I take my tablets with the whisky…and then I might have some breakfast. (8, male, 74yrs)

Since drinking had become a solitary activity for some, getting out of the house could be seen as an important measure in reducing or avoiding consumption. One woman who no longer allowed drink in her house explained:

I used to go out to begin with, that was when I started drinking, and then I started staying in. That was when I started on the vodka because I thought well I’m in the house, if I get drunk nobody can see me. I can get as drunk as I want. I can make as big a fool of myself as I want. I’ve only got to get upstairs to bed, I haven’t got to walk up any banks or owt [anything] like that, and it just went downhill. (16; female, 52yrs)

In contrast to the individual interviews which were personalized, focus group participants tended to present themselves as responsible drinkers but described ‘other’ middle aged or older people they knew or knew of whose drinking was excessive or dependent.

M: I knew a bloke who used to go in the [name] club, and he was, when he used to go in he’d say “I stand here, seven nights a week”, thinking he’s clever. Do you know where he is now? He’s got a leg off [amputated], aye he’s in a home, and he doesn’t know anybody, and it’s all down to drink. (Focus group 2)

Some participants in particular described quite strict drinking habits or routines, for instance meeting particular groups of friends in pubs or clubs on specific nights, to help minimize the chance of heavy drinking. Those still working or volunteering during the week described drinking only on weekend nights when they would not be working the next day, or spoke of *‘waiting till the sun passes the yardarm’*:

F: If it was after six in the evening I'd probably offer a drink before I would offer a tea or a coffee. (Focus group 3)

Routines included a drink out with a meal at lunchtime in the afternoon or merely a ‘*nightcap*’. For some participants, special occasions were the only times they reported having a drink. There were also suggestions that when away from home or on holiday, participants might drink greater amounts or more frequently:

F1: It's relaxing, a glass of wine, it's what you do abroad isn't it?

F2: It is.

GW: Is it? Do things change once you're on holiday?

F3: Oh yes. Totally different on holiday.

F2: Oh I think everybody changes when they're on holiday.

F4: I drink more on holiday, well I do, I drink a lot when I go on holiday. (Focus group 3)

### Deciding to change

Interviewees described consumption in pints, bottles, or cans rather than units and while some were aware of the UK government’s recommended guidelines they tended to be skeptical regarding their helpfulness. It was frequently argued that guidelines would not be relevant to everyone because they did not take account of individual differences in tolerance:

I mean nobody goes out for a drink and says ‘I wonder how many units is in this? I better not drink that’ you know–nobody’s going to say that. (11; female, 55yrs)

Interviewees described themselves as aware of health campaigns targeting drinking, indeed some complained of their ubiquity. For some, public health messages were perceived as class-based ‘preaching’:

I think the random, the average adult doesn’t drink too much in this country, but I don’t know, because I’ve got no figures and I think figures are done, are; they are created, put together by middle class people who haven’t worked at the coal mine or had a fishing boat or in the shipyards and they don’t see the socialisation, the outcomes of it, they only see the bad side of it. (21; male, 56yrs)

It was acknowledged that pressure from family or friends could build the impetus to change drinking behaviour. Older interviewees living in sheltered accommodation described censure from family members if they were thought to have been drinking too much, even though these relatives gave them bottles of alcohol as presents. Interviewees younger than 60 described realising that reducing their drinking might alleviate conflict in their family; for instance having received ultimatums from partners and children:

Then one night he took the kids away from me and I pulled myself together overnight. I just got up one morning and said ‘enough’s enough–I want my kids back’ because my kids were my life. (2; female, 59yrs)

Some younger interviewees had sought help when their partner pointed out the impact of their drinking on the family. One interviewee described his carer as ‘bossy’, but was going to attend counselling because she had persisted in suggesting he do so. Family pressure was not always in the direction of reducing alcohol consumption; if other family members drank dependently, they could be seen as likely to increase the interviewee’s own alcohol consumption. Some interviewees recalled that their family had left them to their drinking, or that it had not been discussed when they came to visit. Some interviewees described themselves as disinclined to drink to excess because of anxiety around alcohol dependency. They recounted that acquaintances or family members had become dependent on alcohol or died from it, and this had made them cautious about drinking.

Concern about the impact of alcohol on older bodies was voiced as likely to make older people reduce their drinking. Amongst those aged under 70 years, hangovers and the time ‘lost’ to them were seen as getting worse with age, and the desire to avoid them strengthened. Other strong incentives to limit consumption for this age group included ambitions for foreign travel and a desire to live more healthily, particularly to ensure a longer relationship with grandchildren or great-grandchildren. For interviewees aged 70+, fear of falling and the desire to avoid gaining weight were influential concerns. These incentives to reduce drinking were raised by both male and female interviewees.

However, when interviewees talked about giving up alcohol, they also recalled varied concerns about the consequences of *not* drinking, such as the loss of an enjoyable part of their lives, possibly the only one left to them:

I’d like to cut it down altogether, but even the last time–I cannot be going away and just sitting on my own. I don’t smoke, I don’t have nowt else and that’s the only pleasure I’ve got–drinking. (4; male, 61yrs)

Dependent drinkers were concerned about severe withdrawals or that they might be unable to cope with a return or worsening of their mental health problems. One interviewee with chronic obstructive pulmonary disease was concerned that cutting down had led her to smoke more. Others thought it would be difficult to spend time with friends or family if they did not drink:

I couldn’t understand how you can have fun just going out and having a cup of coffee. You think how boring. I felt as though I could open up a lot more, you know, when you’ve had a few drinks. (11; female, 55yrs)

Those who had been dependent but had reduced their drinking cited severe medical events, or a warning from a doctor that this was imminent, as powerful incentives to change; for instance, bringing up blood, a diagnosis of pancreatitis or identification of an enlarged liver. Some interviewees felt it was ‘too late’ for them to attempt to change their dependent drinking:

If you’re an alcoholic or an addict and say you’re 25 you have basically maybe 45 years to go, you know, of life. A person who is say 55 and drinking a lot says to himself ‘ah well I'm 55 now what’s there to look forward to now? Doesn’t matter if I stop drinking’ (6; male, 59yrs)

A number of reasons emerged why middle aged and older dependent drinkers might have delayed seeking help with their problem. For example, some perceived it as something to be dealt with by themselves, they felt able to function while drinking and perceived a strong stigma attached to being a dependent drinker, and they had been unaware or uncertain of what help was available.

Only a few participants from the focus groups correctly specified what the recommended guidelines for alcohol consumption were, and most agreed that calculating units was confusing:

M: I’ve seen it on the TV, so many units; I watched a programme about it on the TV, but I can’t remember the units…but they reckon is more, in theory, what women drink, there’s more in the wine, than what it is for beer. (Focus group 2)

At two of the focus groups, participants suggested that many middle aged and older people would regard advertising campaigns against excessive drinking as targeting those who got themselves drunk or lost self-control when drinking, and therefore not relevant to themselves. Participants were also critical of mixed messages from research reports in the media over whether red wine was beneficial or not.

Some incentives were mentioned by participants for moderating drinking, including diets, driving, the desire to see one’s grandchildren grow up, and the desire not to appear foolish:

M: I could go out and drink 7, 8 pints, but it would be making a fool of myself really, you know. (Focus group 2)

One group suggested that many middle aged and older people were motivated to avoid alcohol or minimise their intake because of fears they might become alcoholic.

### Experiences of primary care

Interviewees who recounted problems with alcohol indicated that General Practitioners (GPs) were the first source of help encountered. Participants may have sought help either for their drinking, or for mental health problems with which the drinking was associated:

At the time I was suffering from depression as well and obviously if you want something, some medication, that’s [GP’s] where you’re going to go. (6; male, 59yrs)

However, asking a GP for help usually meant overcoming a reluctance to do so, or to admit to being ‘drunk all the time’ or having an alcohol problem, which could be uncomfortable or embarrassing. One woman had not managed to face asking her GP for help until a friend took her along. Such interviewees recalled trying to hide their drinking from their GP or play down its extent. Some saw GPs as not wanting to treat drinkers, or did not see drinking as a ‘legitimate’ illness to trouble a doctor with:

But you can’t imagine somebody going in [to the doctor] and saying ‘oh, I’ve got a problem, I’m drinking far too much, what help can I get?’ can you? (12; female, 51yrs)

While some participants felt they had been successful in hiding their drinking others recalled that their GP had asked about drinking because of the smell of alcohol:

He [GP] said ‘have you been drinking?’ and I just said ‘aye, 1 or 2’ he said ‘do you know what time it is?’ I said ‘I’m quite aware of the time’ he said ‘I think you’ve got a problem’. (10; male, 60yrs)

GPs were identified, by some, as a source of pressure for changing behaviour. When they had been encouraging about a reduction in consumption, this was seen as positive. However, GPs could also be viewed as having high expectations of abstinence or drastic reductions that participants might have felt unable to meet. They had usually directed participants seeking help to contact a support service or group, or made a referral for detoxification or a psychiatric nurse rather than offering advice on how to cut down themselves:

But Dr [name] is the type of doctor where he’s so laid back, he didn’t specifically say to me ‘that can happen with your liver–that can happen with your brain–that can happen…’ I didn’t get that information. It was just ‘you’ve got a problem–here’s a card now go and deal with it’. (10; male, 60yrs)

One participant thought that many GPs did not understand problems that middle aged and older people faced with drink, but simply referred them on without necessarily knowing the most appropriate service. Some participants felt that their GPs had tried to prescribe them medication in relation to their alcohol problem or associated mental health problems without listening to them or considering alternatives to medication.

No clear differences emerged in experiences of primary care between men and women. Among the oldest group of interviewees (aged 70+), all but one said their GP no longer asked about alcohol; one explained this was “because they know I don’t drink”. The one man who said his GP still asked him about alcohol attributed this to his having had a stroke.

At all three focus groups, participants thought that it was important for doctors to be able to ask about all areas of health, but one group agreed that doctors were overly prone to look to alcohol consumption to explain the symptoms they visited about. Older women in sheltered accommodation on the other hand said that they were never asked, or felt that their doctors knew them well enough personally to know that they had no problems with alcohol. As one woman put it ‘they [doctors] have never had to pick me off the ground’.

### Experience of detoxification and rehabilitation

Most interviewees who reported a serious alcohol problem had been through a detoxification programme at least once, in many cases several times, usually inpatient programmes or emergency responses to a hospitalisation. One interviewee who did not view himself as having any alcohol problems had been outraged to find that he received detox medication when hospitalised for a stroke:

I says ‘get the forms, I’ll sign myself out’. I says ‘all you are thinking about is me being an alcoholic’… They were injecting something into my stomach. (24; male, 72yrs)

Some interviewees had been unable to arrange for a home detox because they lived in temporary accommodation or did not have 24 hour support. Others found it had been difficult to secure a referral for detox because they were not drinking enough at the time, including one woman who said she had been told to reduce her drinking to one bottle of cider a day, but was then told she drank too little to qualify.

Detox programmes had enabled problem drinkers to stop drinking and, for instance, start eating again, but this had almost always been a temporary change. For one interviewee, the abstinence achieved had always been succeeded by complacency and relapse some months down the line. More often participants complained that after detoxification, they had returned to the settings or problems that had fueled their drinking with little or no follow-up to help them avoid alcohol:

Detox and then back to your normal life. I mean the nurses come out every day and they give you vitamin injections which last 6 month[s]. Then come the end of the week, that’s it: they’re gone and you’re back to just keeping away from the pub. You know what I’m saying? It’s hard. (16; f, 52yrs)

Some complained that no attempt was made to address mental health problems alongside a detox. Those referred for detox by counselling services did feel they had been supported through counselling before and after, and appreciated this. One man, for instance, felt that the programme he was about to undertake was more likely than his previous referral to make a difference because of the ten weeks of counselling to prepare him for it.

Rehabilitation programmes were viewed as more expensive, and referrals to them as harder to secure. This required one to demonstrate likelihood to achieve and sustain abstinence. One interviewee did not think that the rehabilitation facilities available were necessarily appropriate for middle aged or older people, describing one establishment as ‘scary’ as it accommodated ‘real winos’. Few participants mentioned having been through rehabilitation, but those who had, described it as an important part of their recovery. Views differed on the usefulness of the group work and activities that formed part of the programme, but the experience of sustained support and individual advice away from everyday routines was seen as a positive factor. They felt again that the support they required during the period after they left the programme had not always been available:

It’s when you come out and you’ve got nobody–that’s when you need them. I came out…I went in on the 30th November, and I was only away a week…I think it was the second week in January I got a letter to say ‘make an appointment to see me’. (11; f, 55yrs)

The lack of support to follow up rehabilitation made relapse likelier, and one woman estimated she had been through five programmes. Long waiting lists were also viewed as a problem, meaning that the impulse or readiness to make a change in behaviour might have passed by the time a place became available.

Due to the smaller numbers of participants experiencing detoxification and rehabilitation there were no differences that could be meaningfully attributed to age or gender.

### Experience of counselling and therapy

Those who had given up drinking as a response to counselling were positive about the help they had received; those who were still drinking and had received counselling appreciated the chance to talk to someone in private but did not see it as having had a significant effect. Counselling was seen as helping to deal with underlying problems, preparing them for other interventions, or supporting abstinence and relapse prevention. One participant whose GP had referred her to a psychotherapist for her mental health problems described this help as ‘brilliant’, as it had helped her understand why she drank. However, the experience of turnover in counsellors, when one moved to a new job, was unsettling, making participants feel as if they had to start from scratch in developing a relationship with the new counsellor, and one participant was inclined not to try counselling again because of this.

Some participants expressed a preference for services that were not specifically dealing with alcohol problems, partly because they felt alcohol services brought them into contact with alcoholics who were likely to encourage relapse; for instance one interviewee said of Alcoholics Anonymous:

It’s just a load of sob stories. I think ‘well I’ve got enough of my own’ without listening to somebody else’s. It made me feel depressed and I wanted a drink when I came out. (11; f, 55yrs)

Groups tended to be appreciated more when they were not focused solely on drinking. Relaxation, alternative therapies and arts classes were popular, helping to fill the day and allowing interviewees to expand their social circle. Both activity groups and drop-in centres (Drop-in centres, generally run by voluntary organisations and local councils, offer emotional support, companionship and practical advice to vulnerable people who live independently in the community) were enthusiastically endorsed as forms of support that gave access to other people to talk with on an ad-hoc basis:

They know where you’re coming from and they're volunteers and they sit and they talk to you and you just chat and it's a way of putting in your time without going to the boozer or getting a drink. (6; m, 59yrs)

It was frequently emphasised that support was best coming from people at the same stage of struggling with alcohol as themselves, or from an ex-alcoholic, in part because they were seen as less likely to be judgmental about drinking and relapses. A voluntary organisation staffed by recovering drinkers was praised because volunteers understood the problems involved but presented the example of health and stability to aspire to. They were seen as a ‘little family’ with which interviewees could connect in the face of isolation experienced elsewhere:

My world is drink and people who drink. The people in the [shopping centre], they might as well–either I might as well not be there or they might as well not be there because I can’t equate with them. (15; m, 65yrs)

Doubts were often expressed about people who had not been through alcohol problems themselves offering help because they lacked first-hand knowledge. Some people were also critical of receiving help from people much younger than themselves who did not know what alcohol problems were like for middle aged or older people, though others thought the age of the person helping you made no difference.

Due to the smaller numbers of participants experiencing counselling and therapy there were no differences that could be reliably attributed to age or gender.

## Discussion

Middle aged and older people’s alcohol misuse may be covert, isolated and carefully regulated and is therefore unlikely to be as obvious as that of young people drinking in public or in external networks of friendship or work groups. Targeting of health messages is therefore important. However the people who we spoke to were skeptical about recommended guidelines for alcohol and rarely measured consumption in units. This is consistent with the literature that older people are one of the least well informed groups about alcohol units both in the UK[30] and Australia[31]. People, in this sample, were also dismissive of the value of health education in line with the lack of evidence regarding effectiveness of such campaigns[30, 32].

Primary health care emerged as important in the identification of problems and provision of advice since this is where middle aged and older people had looked first for help with alcohol problems. This is also consistent with previous research which showed that GPs were the preferred first point of contact for adults with alcohol problems[33]. Screening and brief interventions are effective for middle aged and older adults in primary health care[34–41] yet interviewees felt they had been able to conceal their drinking from GPs, or were not asked about it, and there was broad recognition that it would be difficult to initiate talk about alcohol with a doctor. Research has shown that even when GPs are encouraged to screen for alcohol problems they under-deliver health-promoting advice to older people[42] while nurses report avoiding engagement with older people about alcohol use as they worry about depriving them of the social benefits of drinking[43, 44]. Research from the US also shows that primary care physicians may under detect alcohol use disorders among older patients[45]. Primary care professionals should be aware of the need to ask middle aged and older people about their alcohol consumption and of their possible reasons for concealment; support or training for asking and advising middle aged and older people about alcohol should be available if staff feel uncomfortable with this. In particular it has been suggested that there is a need for the training of community nurses to be focused on improving the detection and treatment of alcohol problems among older people[46].

People in this sample who had experienced alcohol dependence felt that detoxification services had been effective at least in the short term. Other research from the US has reported that older people are at least as likely to benefit from treatment as younger people; and they tend to follow treatment regimens more diligently too[47, 48]. However relatively few older drinkers receive referrals to support services[49] which is consistent with the accounts of our interviewees. Our findings also suggest a compelling need to ensure that aftercare in the form of rehabilitation, counselling, or advice is in place to prevent relapse, or to deal with mental health problems. Strong preferences were expressed by some for support to be provided by those who had experienced alcohol problems themselves but not via a designated alcohol service.

This age group may perceive distinct barriers and incentives to seeking help for alcohol. People described feeling ashamed of being seen as an alcoholic, feeling that services regarded them as ‘on the shelf’, and perceiving themselves as too late in life to change or benefit from treatment. They were concerned about how to cope with boredom, isolation and other health problems without alcohol. However most of those we spoke to had reduced their drinking with or without specialist help. The goals of prolonging life and rebuilding family relationships were reported as strong incentives to change. Evidence for treatment regimes for older people suggest that uptake and success of interventions could be enhanced by facilitating understanding that alcohol problems need not represent an inevitable decline, and helping older people to plan for sustaining an active and involved lifestyle[47, 48] Similar but smaller scale qualitative research with older people that were in specialist alcohol treatment services reported that older drinkers had different stressors, precipitating factors and risk factors for relapse than younger drinkers. They also faced a number of unique barriers to treatment and were more likely to remain ‘hidden’ from services[18].

It has been postulated that behaviour is influenced not only by individual-level attributes but also the conditions under which people live[50]. Participants in this research often cited physical, temporal and social structures as influencing drinking behaviour. Alcohol interventions often disregard these contextual factors which can lead to high relapse once an intervention has ended[51]. Social ecology may be a more appropriate way to target excessive alcohol consumption as it explicitly considers factors in the physical environment that affect health status[52] and suggests a shift away from interventions aimed at changing individual’s health behaviour towards comprehensive ecological designs that address the interdependencies between socioeconomic, cultural, political, environmental, organisational, psychological and biological determinants of health[51]. Examples of social and ecological interventions include social norms marketing and restricting alcohol advertising[53].

Differences by age and gender were attributed to codes where all participants could provide an opinion but not to codes where only a proportion of participants had experience. There were subtle differences in results by age but not gender. Going out to drink was a feature of the accounts of younger interviewees while choice of where to drink could be limited for interviewees aged 70 or more by their mobility or other circumstances, for instance if in a care home. While this change in drinking location seemed to be a direct result of the ageing process there is currently a national trend for less on-trade and more off trade alcohol consumption[54]. Concern about the impact of alcohol on older bodies was also voiced as likely to make older people reduce their drinking. Amongst those aged under 70 years, hangovers and the time ‘lost’ to them were widely seen as getting worse with age, and the desire to avoid them strengthened. Despite lower levels of alcohol consumption, more older people are admitted to UK hospitals with an alcohol-related condition than younger age groups[55]. Other strong incentives to limit consumption for this age group included ambitions for foreign travel and a desire to live more healthily, particularly to ensure a longer or better relationship with grandchildren or great-grandchildren. For interviewees aged 70 years and over, fear of falling and the desire to avoid gaining weight were influential concerns. No clear differences emerged in experiences of primary care between different genders however all but one of the interviewees in the oldest group said their GP no longer asked about alcohol. Due to the smaller numbers of participants experiencing detoxification, rehabilitation, counselling and therapy there were no differences that could be reliably attributed to age or gender.

This research contributes to the limited body of in-depth qualitative data regarding the issues surrounding alcohol consumption in mid to later life, highlighting the needs of middle aged and older people in relation to service provision around alcohol and health. While the sample was relatively small and restricted to a single region of the UK it did provide a depth of data which could not have been achieved via a large quantitative sample. It must be acknowledged however that while one of the aims of purposive sampling was to recruit both genders, females were over represented in the focus groups. Nevertheless considerable diversity was achieved in individual interviews with males well represented. Yet it appears that there is growing international recognition of the needs of older people in relation to alcohol consumption[56]. The most recent Alcohol Strategy for England[25] has requested a review of the current alcohol guidelines for adults including whether separate advice is desirable for older adults. In addition alcohol identification and any subsequent brief advice needed has been included in NHS Health Checks for adults from age 40 to 75 for the first time. Sections of the Models of Care for Alcohol Misusers (MoCAM) also refer specifically to older people[57]. However while organisational standards for drug and alcohol teams contain special guidelines for services for children and young people and for drug-using parents there are no guidelines for older people[58]. In addition the addictive properties of alcohol and access to specialist care for alcohol problems are not mentioned in the National Service Framework for Older People[59]. Finally it was reported in 2008 that the UK had no designated alcohol services for older people[60] and that there was an urgent need for these. We found only one out of 46 services in this large geographical region provided a specific or tailored intervention for middle aged and older people with alcohol related problems[61].

It has been suggested that tailored screening tools perform better with older people[62] and that treatment should be tailored to the unique needs of older people as it becomes more efficacious when it is not generic[63]. The people we spoke to endorse the need for specific support services, tailored to their health in relation to alcohol, to which health professionals feel comfortable referring older people. Primary care professionals, particularly those based in the community, need training on improving the detection and treatment of alcohol problems among middle aged and older people. There is also a compelling need to ensure that aftercare in the form of rehabilitation, counselling, or advice is in place to prevent relapse, or to deal with mental health problems. Strong preferences were expressed by some for support to be provided by those who had experienced alcohol problems themselves but not via a designated alcohol service. Tailored advice would ideally recognise the different issues that we identified for older and younger old for example advice should focus on hangovers and lost time for those in the younger age bracket, compared with fear of falls, seeing grandchildren and the need for doctors to ask proactively with the older age group. These motivating factors are an important basis for tailoring advice and communicating this to primary caregivers who are best placed to offer advice. For example a World Health Organisation (WHO) report suggests that advice about falls risk should be used to reinforce programmes to reduce alcohol use in older people[64]. Promoting the avoidance of harmful use of alcohol is also highlighted by the WHO in their life-course approach to healthy and active ageing[65]. At a broader level, specific policy in relation to older people’s health and alcohol will facilitate WHO objectives of helping people stay healthy and active even at the oldest ages, and helping those who can no longer look after themselves to live with dignity and enjoyment.
